# Epifluorescence-based three-dimensional traction force microscopy

**DOI:** 10.1038/s41598-020-72931-6

**Published:** 2020-10-06

**Authors:** Lauren Hazlett, Alexander K. Landauer, Mohak Patel, Hadley A. Witt, Jin Yang, Jonathan S. Reichner, Christian Franck

**Affiliations:** 1 Center for Biomedical Engineering, Brown University, Providence, 02912 USA; 2 School of Engineering, Brown University, Providence, 02912 USA; 3 Pathobiology Graduate Program, Brown University, Providence, 02912 USA; 4 Department of Surgery, Rhode Island Hospital, Providence, 02912 USA; 5 Mechanical Engineering, University of Wisconsin-Madison, Madison, 53706 USA

**Keywords:** Cellular motility, Biophysics, Motility, Engineering, Mechanical engineering

## Abstract

We introduce a novel method to compute three-dimensional (3D) displacements and both in-plane and out-of-plane tractions on nominally planar transparent materials using standard epifluorescence microscopy. Despite the importance of out-of-plane components to fully understanding cell behavior, epifluorescence images are generally not used for 3D traction force microscopy (TFM) experiments due to limitations in spatial resolution and measuring out-of-plane motion. To extend an epifluorescence-based technique to 3D, we employ a topology-based single particle tracking algorithm to reconstruct high spatial-frequency 3D motion fields from densely seeded single-particle layer images. Using an open-source finite element (FE) based solver, we then compute the 3D full-field stress and strain and surface traction fields. We demonstrate this technique by measuring tractions generated by both single human neutrophils and multicellular monolayers of Madin–Darby canine kidney cells, highlighting its acuity in reconstructing both individual and collective cellular tractions. In summary, this represents a new, easily accessible method for calculating fully three-dimensional displacement and 3D surface tractions at high spatial frequency from epifluorescence images. We released and support the complete technique as a free and open-source code package.

## Introduction

The production of cellular forces has been shown to regulate many important cellular processes including embryogenesis, cellular homeostasis, and disease and trauma response^1–4^. To investigate cellular and inter-cellular force generation, traction force microscopy (TFM) has become a well-established technique in mechanobiology that quantifies tractions produced during cell–cell or cell–matrix interactions, i.e., cell-imposed forces acting on interfaces between a cell or cells and the microenvironment^5–7^. TFM has been cast in both two-dimensional (2D) and three-dimensional (3D) variants^8–11^, from single cells^8–10,12^ to multicellular clusters and sheets^13–16^. While the original development of TFM started with 2D images acquired from phase contrast and epifluorescence microscopy and accounted for only small material deformations, most recent TFM approaches have the capability to fully reconstruct 3D motion and traction fields in a variety of linear, non-linear, and viscoelastic material systems^6,11,17–19^ using confocal, multiphoton, or superresolution techniques^17,20–22^. Despite the significant variations and evolution of TFM, the technique itself consists of three basic steps: reconstructing a cell-induced displacement field from microscopy images using either single particle or image correlation based techniques, inverting a constitutive relation to connect material displacements to stresses, and computing and visualizing traction vector fields^5–7^.

Almost all cellular traction field reconstructions rely on accurate measurements of the cell-induced material deformation fields, which are typically computed from microscope images of fiducial particles, either embedded in the cell substrate^6,8,11^ or micro-patterned onto the substrate surface^23–25^, and analyzed with either a single particle tracking or image correlation-based method. Digital image correlation (DIC) and particle image velocimetry (PIV) are popular correlation-based techniques in 2D; while digital volume correlation and single particle tracking algorithms are commonly found in 3D investigations^6,7,26–28^. The choice of algorithm often comes down to the type of images acquired (e.g., 2D vs. 3D, fluorescent vs. phase contrast, etc.), image content being tracked (e.g., individual embedded particles vs. intensity patterns), and the spatial resolution required.

Once the cell-induced material displacement fields have been determined, the cell-applied stresses, and thus tractions, can be computed in a number of ways. In the early days of TFM, most traction reconstructions relied on the inverse formulation of the classic linear elasticity solution of a point force applied to an elastic half-space. The Boussinesq solution was used to compute tractions initially in real space using the boundary element method^8^ and later in Fourier space (so called Fourier transform traction cytometry, FTTC)^12^. Methods for computing tractions have evolved from these early two-dimensional reconstructions with advancements in imaging modalities and high throughput computing; currently, three-dimensional traction forces can be computed in various ways, including direct solution of the governing constitutive equations^10,21^, using FTTC^29^ or the finite element method^9,17,30,31^. Finite element method traction force microscopy (FEM-TFM) traditionally imposes the measured displacement field onto a pre-defined grid, or mesh, of small elements representing the substrate, and then solves for tractions using a system of equations defined by the mesh discretization of the solution domain^6^. FEM-TFM has greatly expanded the capabilities of 3D-TFM measurements by accounting for complications such as finite gel thickness^9^ and cells fully embedded in a 3D matrix^17,32,33^. It is noteworthy to point out that reconstruction of cellular traction fields using any of these techniques invariably relies upon knowledge of matrix material properties (e.g., Young’s modulus and Poisson’s ratio or shear and bulk moduli) and of the boundary geometry between the cell and the extracellular material (e.g., the hydrogel). Material properties can be determined using a variety of soft-material mechanical characterization techniques while the boundary geometries need to be supplied by the end user either as part of the inverse formulation or through explicit microscopy image segmentation. Several reviews exist that compare the various displacement measurement and traction reconstruction techniques, including their specific applications and advantages and disadvantages^5–7,11^.

Advances in 3D-TFM techniques have revealed that cells produce large deformations into or out of the plane of the substrate^21^, as well as rotational moments about focal adhesions^34,35^. This combination of shear and normal force information reveals substantial new insight into cellular behavior, including how force production is tied to intercellular machinery, how this machinery interfaces with the cell substrate, and how cells turn force production into motion: information that would not be readily available from 2D-TFM. Unfortunately, most 3D-TFM techniques rely upon full-field 3D displacements, which in turn rely upon fully three-dimensional imaging modalities such as confocal or multiphoton microscopy. The availability of these more advanced imaging systems is often limited by prohibitively high costs, which constrains many TFM users to epifluorescence or phase contrast imaging. Due to the native limitations of these traditionally 2D imaging systems, namely the increase in out-of-focus light scattering, those seeking to use these systems for TFM often use a single layer of fluorescent microbeads as fiducial markers to limit out-of-focus light from particles randomly dispersed throughout a gel^36,37^. This adaptation results in planar displacement data, which makes reconstructing 3D stresses for computing tractions challenging. Del Alamo *et al*. demonstrated that using a thin top layer of beaded substrate to limit out-of-focus light scattering in spinning disk confocal images was sufficient for accurately capturing 3D displacements and, in conjunction with 3D Fourier TFM methods, fully 3D traction fields^29^. Until recently, epifluorescence or phase contrast images limited users to in-plane, or two-dimensional, traction information. Makarchuk *et al*. used holographic traction force microscopy to localize and track fiducial particles embedded within the substrate from phase contrast images^38^. In a similar technique, Hall *et al*. used epifluorescence along with a three-dimensional defocused particle tracking method to reconstruct displacement fields from fluorescent fiducial particles embedded in a substrate^39^. These techniques were shown to be effective at localizing 3D bead displacements with high resolution at low spatial frequencies; however, due to the high degree of light scattering native to phase contrast and epifluorescence images, are not able to resolve particles at higher densities and thus are unable to acquire high spatial frequency three-dimensional information.

Here we introduce a technique for computing a fully three-dimensional displacement field and both in-plane and out-of-plane traction components from epifluorescence images of a single layer of beads using a combination of deconvolution, single particle tracking, and finite element analysis. A single layer of fiducial particles limits out-of-focus light from the fluorescent fiducial particles, and enables a high in-plane particle density, allowing us to obtain high spatial frequency information. Our topology-based single particle tracking (TPT) algorithm^26^ allows us to accurately reconstruct high spatial frequency, three-dimensional displacements from the dense layer of fluorescent fiducial particles. The single-plane 3D displacement measurements from the particle tracking algorithm are directly used in a finite element analysis to solve for the full-field, 3D volumetric displacement and stress field quantities, from which surface tractions are computed. By relaxing imaging requirements for 3D-TFM to epifluorescence microscopy while maintaining three-dimensional measurements and high spatial frequency capabilities of traditional 3D-TFM, we aim to enable a broad segment of cell biologists to investigate mechanobiology. To this end, we also provide to the community a new open source, predominately Matlab-based implementation of our technique (see https://github.com/FranckLab) with a detailed discussion of the practical aspects of realizing the experiments in the laboratory available in the Supplementary Material.

## Results

**Figure 1:**
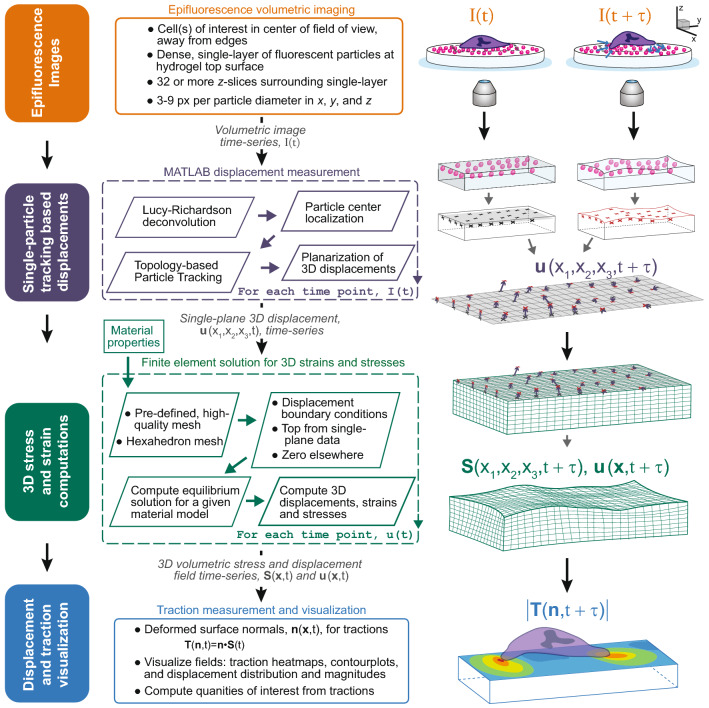
The procedure for calculating tractions from epifluorescence imaging. Volumetric epifluorescent images of spherical fiducial markers (i.e., fluorescent microbeads) embedded at the surface of a cellular substrate medium are imported into Matlab where they are deconvolved and a particle tracking algorithm is employed to calculate 3D displacements. The nearly-planar scattered displacements are mapped onto a planar grid with a second order lookup-table regularization. These 3D surface displacements are then input into a finite-element based solution to the boundary value problem defined by the known and measured displacement fields and material properties. From this computation, the stresses and a full-field 3D displacement field in the substrate domain are extracted and used to compute in-plane and out-of-plane traction components. The tractions may then be used to compute biologically-relevant quantities of interest. The right-hand column provides a simplified illustration of the process for images taken at time, *t*, and after some time, $\tau$.

We present a new method to obtain 3D displacement and surface 3D traction fields from epifluorescence images. The complete procedure is outlined in Fig. 1 and the description of the method is broken down in more detail in the remainder of this section. To summarize these steps, 3D volumetric image stacks of cells on a planar substrate with a single layer of fluorescent microbeads at the surface are taken on an epifluorescence microscope. These are imported to Matlab, where the images are deconvolved, and particles localized and tracked using topology-based particle tracking (TPT). The resulting nearly planar layer of measurement locations are used to project the displacement onto a planar grid to define a displacement boundary condition before being output to the free and open source finite element analysis (FEA) program FEniCS^40,41^, where the solution for the full-field displacement and stress information is computed. This full field information is then imported into Matlab for final traction computation and visualization.

### Single-layer bead polyacrylamide substrate fabrication

**Figure 2:**
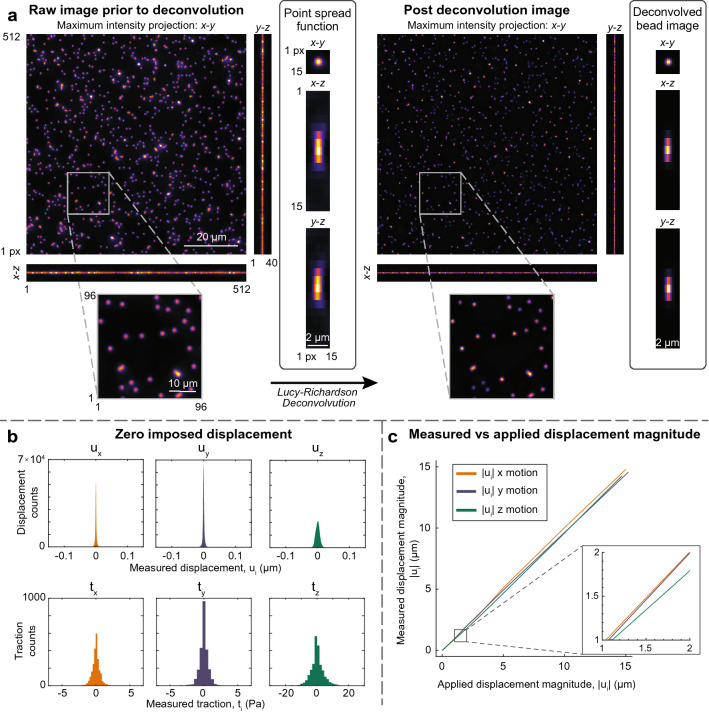
Typical single-layer beaded gel images from epifluorescence microscopy and resultant displacement reconstruction accuracy metrics. (**a**) Maximum intensity projections from both pre- and post-deconvolution results for an experimental image using the experimentally-gathered point spread function shown in the center column. The deconvolved point spread function is shown in the column on the right. Insets show a closer view of the maximum intensity *z*-projections of experimental images pre- and post-deconvolution. (**b**) Histograms showing the distributions of the reconstructed planarized displacement in *x*, *y* and *z* from a nominally zero-displacement experimental image pair and resultant *x*, *y* and *z* tractions. Note the zero-centered symmetric nature, indicating little bias or skew at the noise floor. (**c**) Line plot showing the measured mean displacement magnitude versus the applied experimental displacement magnitude from three individual rigid body displacement experiments, each imposing rigid motion in one of the three principal directions with sequentially increasing displacements along the indicated axis. Standard deviation is plotted as a shaded error bar (very small); inset shows the shaded error bars in more detail.

Polyacrylamide gels with a single layer of fluorescent microbeads were created using a modified version of the protocol previously described by Knoll *et al*. (2014)^36^. Briefly, beads were adhered to a poly-l-lysine coated glass coverslip, which was then used to sandwich a droplet of polyacrylamide gel solution during polymerization, as originally described by Pelham and Wang (1997)^42^. The bead-coated coverslip was removed, leaving the layer of beads embedded in the gel surface. For more information on polyacyrlamide gel preparation, see the “Methods” section. Representative images demonstrating limited *z*-spread of the bead positions, with beads randomly distributed with uniform density in *x–y* (i.e., limited bead clumping, empty regions, or other inhomogeneities) are shown in maximum intensity projections in Fig. 2a.

### Epifluorescence volume image deconvolution

We measure high spatial resolution displacements using our single particle tracking algorithm. This technique utilizes densely seeded fluorescent microbeads as fiducials in our volumetric images, and requires each bead to be fully resolved at high resolution with Gaussian intensity and a characteristic diameter of approximately three to nine voxels in each direction. To obtain volumetric images of the fluorescent microbeads with these characteristics from epifluorescence microscopy, we deconvolve the experimental images using the point spread function (PSF) collected from a single microbead directly from the experimental images. To do this, we choose an isolated microbead and select a sufficiently large region of interest surrounding the bead in all three dimensions to capture the light spread from only the isolated bead. An example of a microbead PSF extracted from an experimental image can be seen in the single-bead maximum intensity projection in Fig. 2a. Further imaging details are given in the “Methods” section. Under the assumption that each microbead is a point-like light source, the single bead image thus describes the transfer function of light from a point source to the detector. Under these assumptions, we have found a straightforward application of the maximum likelihood estimator-based Lucy–Richardson deconvolution scheme^43,44^ sufficiently repeatable to obtain bead images that admit accurate localization in *x*-, *y*-, and *z*-directions; see Fig. 2b and c. It is, however, noteworthy to mention that images should be taken with care to avoid skewness and severe imaging artifacts, which substantially degrade reconstruction and subsequent fiducial marker localization and tracking performance.

To compute the deconvolution, we use the Lucy–Richardson type scheme that assumes the transfer function of the optical system is applied to the true bead image in the presence of shot noise and background fluorescence (i.e., additive Poisson and Gaussian noise). The deconvolution proceeds by solving for the pristine image estimate via an iterative inversion approximation of the transfer function plus noise^45^. We have employed the more sophisticated implementation of this algorithm built into the Matlab Image Processing Toolbox. Pre- and post-deconvolution volumetric image reconstructions are given in Fig. 2a and show the reduction of apparent particle size in the *x*–*y* plane, which aids precision, and results in a dramatic reduction in *z*-spread, which enables sufficient precision and accuracy in the *z*-direction localization for effective out-of-plane deformation measurement.

### 3D displacement field from single-particle tracking

Our topology-based particle tracking (TPT) algorithm is used to reconstruct high-spatial frequency, large deformation displacement information from the deconvolved images. To begin, the bead centers from deconvolved bead images are localized to determine bead positions with subpixel accuracy via the radial symmetry method, described by Liu *et al*. (2013)^46^, as implemented in TPT. Then, for each image time point the regional particle location data are used to build a feature vector describing each bead in the image. For each bead, TPT links these feature vectors between reference (undeformed, $t = 0$) and deformed ($t = t + \tau$) images to determine a 3D displacement vector. By employing the iterative deformation method, link verification, and displacement outlier removal schemes, a relatively simple feature vector is used to track large, high spatial frequency displacements and provides a computationally efficient means to fully resolve 3D displacements at single particle resolution. For our nominally single-layer gels all beads found and successfully linked within the image volume are tracked, including spurious out-of-plane beads, resulting in a scattered loci of measured displacement vectors. The subsequent planarization step both selects displacement data only on the dominant top single layer of beads and resamples the displacements onto a regular grid, see below. In the future, globally-aware tracking and linking, e.g., via an augmented Lagrangian-type regularizer to guarantee the global kinematic compatibility^28^, a mesh-free type interpolant^39^, or more computationally demanding feature vector could further improve the quality of tracked 3D displacement fields.

### Planarization of 3D TPT data

Displacement data recovered using TPT are located at bead coordinates within the volume, and thus are scattered. Outlier beads are rejected from the tracking result using a user-tuned parameter for the number of standard deviations that an individual bead lies from the overall mean bead *z*-height. Outliers are uncommon using our single layer bead technique and thus are typically isolated and suffer from increased noise. In addition, to impose the top-surface Dirichlet-type boundary condition on the initially flat computational volume, displacements must reside on a single *z*-plane. To address this, displacements are resampled onto a regular grid located on the best-fit plane of bead locations; this grid is the top surface mesh that will be used in subsequent 3D finite element simulations, thus also correcting for any tilt of the stage or gel surface with respect to the imaging coordinates. The displacement resampling is performed by a curvature regularization scheme of the scattered bead displacements, with user-adjustable smoothing coefficients for the regularizer implemented independently between (*x*-*y*) and *z*-displacements. This leads to less noisy and more robust displacements ($[u_x, u_y, u_z] \in$ Hilbert space $H^{2}$) for use in determining volumetric stresses and strains via a forward solution of the boundary value problem with finite elements. Figure 2b and c show the distribution and accuracy of planarized displacement information from a zero-displacement rigid body motion experiment and the measured displacements plotted against the applied rigid displacement for three rigid body translation experiments (one in each of *x*-, *y*-, and *z*-directions), respectively. These demonstrate that this technique provides a level of accuracy and precision necessary to compute stress fields for small, low force-producing cells such as the human neutrophil.

### 3D finite element calculations for strain, stress, and traction

**Figure 3:**
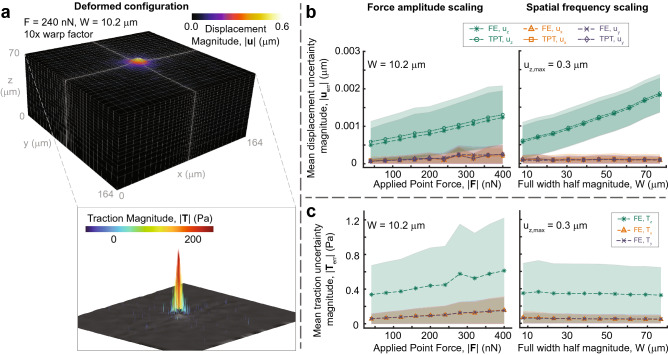
Finite-element set up and uncertainty for a simulated case consisting of images synthesized with independently varied traction magnitude and width. (**a**) The final converged hexahedron mesh in the deformed configuration with refinement at the center of the applied Gaussian traction field. Displacement magnitude contours are shown on the the deformed mesh, to which a 10× deformation amplification has been applied to aid visualization. (*Inset*) Measured surface traction vector field for this example case plotted on the deformed configuration top surface. (**b**) Displacement uncertainty magnitude for synthetically generated Gaussian-profile tractions with varying applied force amplitude and spatial frequency (full-width half-magnitude of the Gaussian traction profile, with fixed peak resultant displacements). (**c**) Traction reconstruction uncertainty projected onto the undeformed surface (i.e., normal and two in-plane traction vectors) for the same test cases. The shaded regions reflect the pointwise standard error for each case.

We directly solve the volumetric boundary value problem defined by known, measured displacements and substrate material properties using the finite element method as implemented in FEniCS. A baseline, convergent finite element mesh (tested via a synthetic 6$\sigma$-width Gaussian indentation displacement with amplitude approximately 0.27 μm), consisting of 25,992 hexahedron mesh elements (i.e., first order Lagrangian bricks^41^) is a baseline for all experiments, see Fig. 3a. The mesh is automatically pre-refined in the region of the cell according to an adjustable Gaussian decay in element density. This initial mesh provides a consistent, high resolution, and high quality starting point on which to apply the TPT-based planarized displacement and solve the volumetric boundary value problem. Linear hexahedron elements with center nodes are used to avoid locking, given the relatively large Poisson’s ratio of typical hydrogels used for TFM. To accommodate varying experimental configurations, the base mesh is uniformly warped to fill the image volume, as defined in the output from the Matlab-based pre-processing steps detailed above. Since the base mesh is a regular rectangular solid and most imaging domains are similar in shape and aspect ratio to this mesh, distortions from this reshaping are typically negligible. As with planarization, more sophisticated meshing (e.g., adaptive remeshing) is possible, but we have obtained efficient and robust solutions with acceptable accuracy using the straightforward approach described above. Regarding displacement boundary conditions, the left face of the computational volume is fixed in the *x*-direction (i.e., $u_x = 0$), the back face is held fixed in the *y*-direction (i.e., $u_y = 0$), and the bottom face held fixed in the *x*, *y*, and *z*-directions (i.e., $u_i = 0$), thus fully constraining the solution domain. The top surface displacement boundary condition is given by our planarized 3D cell-imposed displacement vectors output from the preceding analysis steps. The validation of this technique (see the “Synthetic validations” section in the “Methods” and Fig. 3) demonstrates that small, subpixel displacements (i.e., those generated by tens of nanonewton cellular forces) and high spatial frequencies (i.e., micrometer-scale full-width half-magnitude (FWHM) frequency) are observable with signal to noise ($\text {SNR} = \frac{\text {|Uncertainty|}}{|\text {Signal}_{\text {max}}|}$) ratios of approximately 300 and 100 for displacement and traction, respectively.

The stored strain energy density function, $\Psi$, used to define element-wise constitutive relations is given by the compressible form for the neo-Hookean hyperelastic solid, i.e.,

1 $$\begin{aligned} \Psi = \frac{\mu }{2} \left( {\text {Tr}} {\mathbf {C}} - 3\right) - \ln J+\frac{\lambda }{2} (\ln J) ^2, \end{aligned}$$

where material properties μ and $\lambda$ are the Lamé constants computed from user-input elastic modulus (E) and Poisson’s ratio ($\nu$) of the hydrogel. The tensor-valued ${\mathbf {C}}$ and scalar-valued *J* terms represent right Cauchy–Green deformation tensor and volume change ratio of a generic finite-strain deformation of the element from a reference (undeformed) to deformed configuration and ${\text {Tr}}(\cdot )$ is the trace operator (see, for example, the textbook of Bower^47^ for a complete discussion of large deformations in continua). In short, we take ${\mathbf {C}} = {\mathbf {F}}^T{\mathbf {F}}$, where ${\mathbf {F}}$ is the spatial deformation gradient of the displacement field and $J = {\text {det}}({\mathbf {F}}) > 0$ where ${\text {det}}(\cdot )$ is the determinant operator. Stresses are projected on each element in the volume via second-order quadrature of the hexahedron elements.

The traction vector field on the top surface, which is currently assumed to be wholly due to the action of the cell, is computed from the deformed configuration of the finite element mesh and true stress projected from quadrature point to the deformed nodal coordinates of the mesh. To determine traction at each point on the top surface the Cauchy relations are computed via

2 $$\begin{aligned} {\mathbf {T}}({\mathbf {n}}) = {\mathbf {n}} \cdot \mathbf {\sigma } \end{aligned}$$

where ${\mathbf {n}}$ is the surface normal vector, $\mathbf {\sigma }$ is the Cauchy (true) stress, and ${\mathbf {T}}$ is the traction vector. Surface normals are determined via a Delauney triangularization of the deformed top surface of the measurement volume and the stress field interpolated with a natural neighbor interpolant (C$^1$ continuous except at sample points) onto the vertex points in the trangularization (viz. the method of Toyjanova *et al*. (2014)^21^).

### Computing 3D cell traction fields

**Figure 4:**
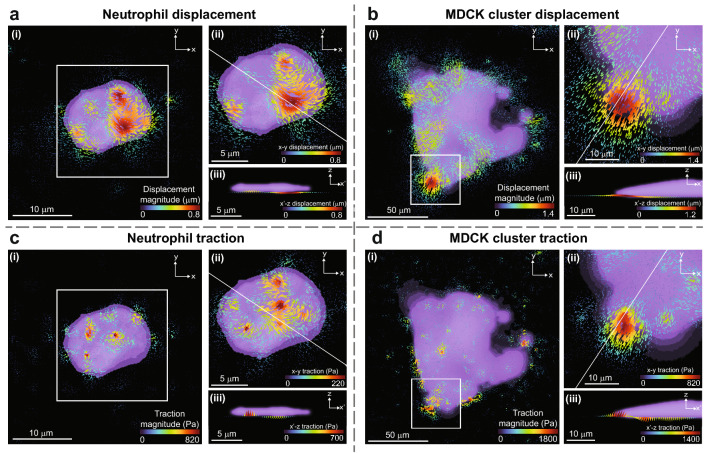
Cell-produced 3D displacement and traction fields on the top surface of the hydrogel. Maximum intensity projections of the cell bodies are shown in purple. Cone size and color imply displacement or traction magnitude, while direction is given by the cone points. (**a**) The displacement field visualization for an activated neutrophil migrating on a fibronectin coated polyacrylamide hydrogel showing localized peak displacements toward the cell center and downward into the gel. (**b**) The displacement field visualized for a multicellular cluster of MDCK cells during collective migration along a collagen-I coated polyacrylamide hydrogel displaying contractile displacements around the entire cell cluster. (**c**) The resultant surface traction vector field for the neutrophil case computed on the deformed top surface reconstructed in the finite element analysis. The traction distribution exhibits a push-pull pattern often associated with amoeboid cell migration. (**d**) The traction for our MDCK cell cluster. Disperse tractions are centered on a downward (into the gel) push near the middle of the cluster. For each case, (i) shows the *xyz*-magnitude, (ii) shows a magnified view of the *xy*-magnitude of the area within the white box shown in (i), and (iii) shows the *xz*-vector of the displacement or traction field projected onto the *x*$\prime$–z cutting plane, i.e., a cutting plane view of the vertical slice made by the white line shown in (ii).

We apply this procedure to compute 3D traction fields from two cells types to demonstrate its effectiveness in practice. Images of neutrophils and Madin–Darby canine kidney (MDCK) cells labeled with Calcein AM fluorescent dye were acquired using epifluorescence and 3D boundaries were segmented using built-in Matlab functions for image thresholding and binarization. Phase contrast images of cells could also be segmented and binarized to be used for plotting planar information but are not typically sufficient for segmenting and plotting three-dimensional cell boundaries, such as those shown in Fig. 4a–d(iii). An example of the fluorescence cell images acquired is shown in the Supplementary Material. First, we examine a single primary human neutrophil. Neutrophils have been shown to exhibit various traction patterns using TFM, including concentrated forces in the uropod of the cell, differential tractions on different substrate stiffnesses, and large out-of-plane deformations, among others^21,48,49^. In addition, neutrophils are both small, typically 8–10 μm in diameter, and fast-moving, thus demonstrating the ability of our technique to quantify high temporal and spatial frequency displacement and traction information. An example of a three-dimensional displacement field from a human neutrophil can be seen in Fig. 4a. The 3D displacement magnitude is shown in Fig. 4a(i), the *x–y* displacement of a cropped region is shown in (ii). To more clearly show the measured three-dimensional deformation for a region of interest within the volume, the displacement distribution is shown in (iii) as a projection onto the arbitrary cutting plane *x*$\prime$–*z*, i.e., a vertical cutting plane selected along a vector *x*$\prime$ in the *x*–*y*-plane such that the points of interest in the cell are transected. The corresponding traction field is shown in Fig. 4c, with the magnitude, *x–y*, and *x*$\prime$–*z* tractions shown in (i), (ii), and (iii), respectively. Second, we demonstrate that our technique can be used for not only single cells, but also multicellular monolayers or two dimensional cell clusters. For this case, we chose MDCK, which are commercially available and have been shown to produce quantifiable traction forces during collective migration, and as individual cells or in groups or clusters^14,50,51^. An example of the three-dimensional displacement and traction fields produced by a cluster of approximately 20 MDCK cells can be seen in Fig. 4b and d, respectively, in a layout identical to that described for the neutrophil case.

## Discussion

The procedure described in this paper represents a new technique for calculating three-dimensional displacement, stress and surface traction fields from epifluorescence images of fluorescent fiducial particles embedded in a two-dimensional surface layer and acted upon by an external force, such as a cell or cell cluster. Using deconvolution with an experimentally determined point spread function, the particle centers are accurately localized in the *z*-direction. The deformation is reconstructed with a single particle tracking algorithm (TPT) that excels at reconstructing high-spatial frequency motions in high particle density environments. The tracked particle displacements are then resampled onto the best-fit plane of the initial bead locations. This planarized single-layer displacement information is then applied as a surface Dirichlet-type boundary condition in the FEniCS finite element solver. With user input material properties (elastic modulus and Poisson’s ratio) given for the substrate, the full 3D displacement and state of stress of the substrate volume is directly solved assuming a hyperelastic neo-Hookean material model. The resultant top-surface tractions are computed using finite deformation continuum principles^21^ and, finally, complete visualizations of displacement and traction fields are rendered.

We validated and tested this protocol using a number of synthetic and experimental cases to probe the accuracy and precision of the reconstructed displacement and traction fields, and to examine its capacity to reconstruct high spatial frequency motion fields. We also demonstrate the technique on two cellular cases. To assess the accuracy and precision of the process for measuring displacements in real experiments, rigid body motions with displacements ranging from 1 μm to 15 μm were imposed and reconstructed. Spatial frequency, amplitude, and traction field reconstruction was validated using synthetic Gaussian tractions, which demonstrates the ability of our algorithm to accurately resolve tractions at cell-relevant scales. Finally, we compute traction fields from two cellular examples to represent two different types of data that are commonly analyzed using traction force microscopy: a single cell and a multicellular cluster. This suit of tests demonstrates the ability of our algorithm to reconstruct displacements and quantify tractions from a variety of cell-produced substrate deformations. We chose to compute traction from a cell cluster in our example case, rather than tractions from fully confluent monolayers, to preserve a zero-displacement edge condition^13–15,51,52^, though the technique could be readily extended to compute intercellular stresses of a confluent monolayer of cells, e.g., Serrano *et al*. (2019)^15^.

Our displacement and traction example cases showcase some of the information that is only accessible with three-dimensional TFM analysis. A visual comparison between Fig. 4a–d (ii) with (i) and (iii) shows that the out-of-plane displacement and traction produced often match or exceed the in-plane values. In addition, the three-dimensional analysis highlights the rotational behavior of cell displacement and traction forces concentrated at the edges of the cell or cell cluster initially described by Legant *et al*.^34^. These observations carry across our two very different cell examples, and highlight the importance of using three-dimensional traction force microscopy techniques to make complete observations about cell behavior.

In principle, epifluorescence imaging can be used for three-dimensional traction force microscopy without the need to use a single layer of beads and hence finite element methods for analysis. However, the spatial resolution of the resulting displacement and traction information will be limited by the ability to resolve and adequately localize bead spread in the *z*-direction^39^. That determination will ultimately depend on the reconstructed 3D image quality and the quality of the optical system. Here, a single layer of microbeads allows us to use a high seeding density, and thus resolve higher spatial frequency information without having to directly contend with light scattering from epifluorescence inhibiting our ability to accurately localize out-of-plane microbeads. It is also noteworthy to mention that we have used an inverted microscope to maximize bead image quality and subsequent localization: in upright microscopy, light scattering through the cell or cell layer may result in additional degradation of image quality and therefore localization accuracy. As in the fully 3D volumetric case, mean deformation metrics^18^ of the cell, e.g., mean contractility in the principal directions, could be extracted from reconstructed motion estimates if cell-scale constitutive relations for a substrate material are unavailable. Considerations for viscoelastic or alternative non-linear material model formulations could be straightforwardly incorporated via implementation of the strain energy density function in FEniCS, further increasing the potential applications of this protocol.

The complete Matlab and FEniCS-based implementation of the workflow are packaged with a user manual and are available for download as free and open-source software (see https://github.com/FranckLab). By enabling users to implement an inexpensive and readily available imaging modality to acquire the necessary data, and by releasing a straightforward, documented protocol with example data and all algorithms required for computation and plotting included, we seek to lower the threshold for users to probe three-dimensional cellular mechanics.

## Methods

In this section, we begin by detailing the method for validation and verification of the technique. We then briefly discuss the setup and data collection for experimental error assessment via rigid-body translations of a representative cell-free specimen. Finally, we describe the complete preparation and imaging process for the neutrophil and MDCK cell test cases.

### Synthetic validations

To vet the accuracy of the algorithm with known imposed fields, synthetic images were generated with Gaussian indentation-like traction distributions. In this way, we test the algorithm while independently and self-consistently varying the spatial frequency and amplitude content of the displacement signal over a range of synthesized experiments.

A previously described bead seeding algorithm^26,27^ that places bead images into an image volume at subpixel locations and with minimal interpolation was employed. Undeformed configuration images were generated in a four step process. First, a representative real image of a bead was extracted from an experimental image (see the “Cell preparation and imaging” section). Second, bead center positions were seeded in the volume with random non-overlapping coordinates at the approximate density and spatial distribution observed in experiments. Third, bead images were added to the image volume at the predefined locations, and, fourth, the final images were corrupted with a Gaussian white noise profile and dynamic range adjusted to match measurements from our experimental test cases. To then generate the deformed images the displacements for each seed location were sampled from the analytical solution for Gaussian *z*-traction indentation of an elastic half-space with material properties corresponding to typical experimental parameters ($E=1500$ Pa and $\nu =0.45$) for a series of increasing spatial frequencies, controlled via the full-width half-magnitude (FWHM) of the signal ranging from $W = 7.68$ μm to $W = 76.8$ μm in steps of $7.68$ μm at a fixed 0.32 μm peak displacement amplitude and force amplitudes from $F = 40$ nN to $F=400$ nN in steps of 40 nN at $W = 10.24$ μm. The frequency and amplitude ranges were selected to encompass typical experimental observations, for example those found in Toyjanova *et al*.^53^. For each case, the analytical solution for displacements and tractions are based on a convolutional Fourier-space Boussinesq solution that are simultaneously computed at each measurement point.

The synthetic volume images were propagated through the complete analysis workflow of Fig. 1, and uncertainties were computed for displacements and tractions. Figure 3 shows the mean uncertainty magnitude (see Eq. 3) of TPT displacement, post-finite element solved displacements, and traction magnitude. The finite element mesh with color contours of measured displacement in the deformed configuration (10× warping factor for visualization) is shown in Fig. 3. The uncertainty quantification between the analytically solved and measured synthetic fields is computed using the mean of the pointwise error between reconstructed and imposed ground truth via

3 $$\begin{aligned} |\text {Uncertainty}| = \frac{\sqrt{\sum _n \left( \mathbf {\chi }_{\text {measured}} - \mathbf {\chi }_{\text {imposed}} \right) ^2}}{N} \end{aligned}$$

where *n* are the measurement points (e.g., quadrature points or mesh vertices on the top surface), $\mathbf {\chi }$ is a field quantity (e.g., displacement or traction), and *N* is the total number of measurement points for the quantity. The results from this uncertainty formulation, which describes an approximate non-parametric confidence interval-type uncertainty for a given measurement point in its base units, are plotted independently as functions of signal magnitude or spatial frequency in Fig. 3b and c with the standard error of the measurement shown as shaded area.

### Experimental validation and live-cell experiments

#### Polyacrylamide gel preparation

Glass coverslips were cleaned using ethanol and dried. 0.1% w/v Poly-l-lysine (PLL) was coated onto the surface of the coverslips and allowed to sit for 1 h. Suspended 0.5 μm carboxylate modified polystyrene beads (Thermo Fisher Scientific) were vortexed for 30 s to 1 min prior to dilution to a final ratio of 1:100 in deionized water. Poly-l-lysine was blown off of the surface of the glass coverslips using air, and the treated surfaces were then coated with the 1:100 bead solution and allowed to sit for 30 min before being blown dry again. Glass coverslips were hydrophilically treated using amino 0.5% (v/v) 3-aminopropyltrimethoxysilane in ethanol, followed by 0.5% glutaraldehyde in deionized water. Finally, polyacrylamide gels were fabricated using the method originally described by Pelham and Wang 1997^42^, where polyacrylamide gels are polymerized between two glass coverslips; in this case a hydrophilic coverslip on the bottom and a bead coated coverslip on the top. Gels of two stiffnesses were fabricated at two relative concentrations of acrylamide and bis-acrylamide (Bio-Rad), namely 8%/0.08% and 3%/0.2%, to produce nearly incompressible gels with Young’s moduli of 8.3 kPa ± 0.2 kPa and 1.5 kPa ± 0.1 kPa, respectively. Stiffnesses for this procedure were measured by Toyjanova *et al*.^53^ via an established uniaxial compression technique^10,54^. Crosslinking was initiated with the addition of ammonium persulfate (Sigma-Aldrich) and *N*, *N*, *N*, *N*-tetramethylethylenediamine (Sigma-Aldrich). MDCK cell experiments used 8.3 kPa gels, whereas neutrophil and all other experiments used the 1.5 kPa gels. Gels were given 15 min to polymerize before being immersed in deionized water, and allowed to swell for 45 min. The PLL-coated coverslip was peeled off, leaving the layer of beads embedded in the gel.

#### Single-layer gel imaging parameters

Polyacrylamide gels with a single layer of fluorescent beads were imaged on a Nikon TI-2 epifluorescence microscope using a 40 $\times$/0.6 NA air objective with an aligned correction collar. A three-dimensional stack of epifluorescence images were taken around the beads, with a μm-per-pixel ratio of 0.16 and a *z*-step size of 0.3 μm (or 1.0 μm for MDCK cells), per the recommended Nyquist sampling rate for the chosen objective and system. Raw images of 2560 voxels $\times$ 2156 voxels $\times$ 101 voxels are typical for our imaging system (pco.edge 5.5, PCO AG) and can be used at full resolution in the algorithm on a workstation computer with 64GB RAM, but are cropped to 1024 voxels $\times$ 1024 voxels $\times$ 101 voxels (see Supplementary Material) to reduce computational resources required when the cells of interest reside in a subset of the image, as was the case for our neutrophil and MDCK cell test data shown in Fig. 4.

#### Rigid body displacement validation

To assess the ability of our algorithm to accurately localize and track displacements from epifluorescence images and to create a planar displacement field that matches a known displacement condition, we performed a series of rigid body displacement experiments. This experimental image set was captured by taking an initial image stack with the single layer of beads centered in *z* and imposing a known incremental stage motion in *x*-, *y*-, or *z*-directions before acquiring a new image stack. The stage motion setpoints were 0.0 μm, 1.0 μm, 2.0 μm, 3.0 μm, 5.0 μm, 10.0 μm, and 15.0 μm, and images were captured of the microbeads after the prescribed stage motion in each of the three orthogonal directions. We then interrogate the accuracy and precision with which our method resolves known displacements based upon the read out from the stage encoder (Nikon Instruments, positioning accuracy ± 0.1 μm). We plot the distribution of the reconstructed displacements for a zero rigid body motion case in Fig. 2b, as well as the measured versus applied displacement, for all of the rigid body motion cases, as shown in Fig. 2c. The positional accuracy of the stage encoder is much smaller than imposed rigid motions, (± 0.1 μm vs. minimum 1 μm), and does not appear to be a significant error source based on this displacement analysis.

#### Cell preparation and imaging

For cellular experiments, polyacrylamide (PA) gels were treated with the crosslinker *N*-sulfosuccinimidyl-6-(4$'$-azido-2$'$-nitrophenylamino) hexanoate (Sulfo-Sanpah, Thermo Fisher Scientific Pierce), for two, 15 min increments under UV light, washed with 1 $\times$ PBS, and then coated overnight with either 0.2 mg/mL fibronectin (neutrophils) or 0.2 mg/mL collagen-I (MDCK cells), following established protocols^21^. Excess protein solution was washed off using PBS before the PA gels were incubated at $37\,^{\circ } \hbox {C}$ in imaging media prior to the addition of cells.

Blood was taken from healthy volunteers with written informed consent through a protocol approved by the University of Wisconsin Internal Review Board and in accordance to the guidelines and regulations thereof. Primary human neutrophils were isolated by negative antibody selection using the MACSxpress Neutrophil Isolation and MACSxpress Erthryocyte Depletion kits (Miltenyi Biotec, Inc.). Cells were labeled by 8 min incubation in 2 μM Calcein AM in Hank’s Balanced Salt Solution, without calcium, magnesium, or phenol red. Cells were seeded onto fibronectin-coated PA gels in Leibowitz’s L-15 media with 2 mg/mL glucose added, and allowed to adhere for 20 min at $37\,^{\circ } \hbox {C}$ prior to imaging. Neutrophil images were taken with the same 40 $\times$/0.6 NA air objective as all other images, but with the Nikon TI-2 1.5 $\times$ optical zoom lens in place to help resolve the smaller cells.

Madin–Darby canine kidney type II cells (Sigma Aldrich) were cultured in low glucose Dulbecco’s modified eagle medium (DMEM, Fisher Scientific) and fetal bovine serum until nearly confluent. MDCK cells were removed from culture dishes by incubation with Trypsin-EDTA (Thermo Fisher Scientific) and then seeded onto PA gels coated with collagen-I. The cells were allowed to adhere and proliferate for 24 h, until nearly confluent, prior to staining with 8 μM Calcien AM for 20 min in media, and then imaging.

All live-cell experiments were conducted in a custom-built temperature control chamber at $37\,^{\circ } \hbox {C}$. Three-dimensional *z*-stacks of fluorescent microbeads in the substrate and fluorescently labeled cells on the substrate surface were taken simultaneously to capture displacement information. Following acquisition of images of both types of cells, cells were removed from the PA gels using 5% sodium dodecyl sulfate (SDS). A final volumetric image stack for the reference condition was acquired 15 min after the addition of the SDS, when all of the cells had detached from the gels.

### Computational requirements

Cell image stacks were taken at 2560 px $\times$ 2156 px by 101 slices and cropped to 1024 voxels $\times$ 1024 voxels $\times$  40 voxels around the cell border for analysis on a desktop PC with 32 GB of RAM. Deconvolution, the most memory-intensive step of the process, requires approximately 64 GB RAM for full-size images, which may take several hours per image, but both time and memory usage is significantly reduced for the cropped images. Regarding processing time (Intel i7-4790 at 4 GHz, 32 GB DDR3 at 1600 MHz, NVME M.2 solid state storage drive, synthetic validation test case), deconvolution of the cropped images takes approximately 90 s per image stack. Timing for bead localization and computing displacements closely follows the timing results in Patel *et al*.^26^—approximately 20 s on 3 parallel threads. Regularization is completed in approximately 1 min, and the following plotting and output steps are accomplished in approximately 20 s. By far the most time consuming stage of the computational process is the finite element solver, which is computed with 16 GB and 2 CPU cores allocated for Docker in approximately 10 min per time point image pair. In future, more extensive multi-processor capability could be straightforwardly added if needed. Post-processing to compute surface tractions takes only a few tenths of a second. Thus total runtime (neglecting time for user interaction) may be budgeted at approximately 15 min per time point image pair.

## Supplementary information

Supplementary Information.

## Data Availability

All data used to generate figures and results for this work are freely available and hosted on the University of Wisconsin - Madison MINDS research data storage service at the following web link: http://digital.library.wisc.edu/1793/80302. As mentioned earlier, the code is also freely available from the Franck Lab GitHub repository. Code, data, and assistance are also available by contacting the authors (raising an “Issue” in GitHub is preferred).
