# Publisher Correction: Epifluorescence-based three-dimensional traction force microscopy

**DOI:** 10.1038/s41598-021-83840-7

**Published:** 2021-02-18

**Authors:** Lauren Hazlett, Alexander K. Landauer, Mohak Patel, Hadley A. Witt, Jin Yang, Jonathan S. Reichner, Christian Franck

**Affiliations:** 1 Center for Biomedical Engineering, Brown University, Providence, 02912 USA; 2 School of Engineering, Brown University, Providence, 02912 USA; 3 Pathobiology Graduate Program, Brown University, Providence, 02912 USA; 4 Department of Surgery, Rhode Island Hospital, Providence, 02912 USA; 5 Mechanical Engineering, University of Wisconsin-Madison, Madison, 53706 USA

Correction to: *Scientific Reports* 10.1038/s41598-020-72931-6, published online 06 October 2020

In the original version of this Article, the Equal Contribution statement for the authors Lauren Hazlett and Alexander K. Landauer was omitted.

This error has now been corrected in the HTML and PDF versions of the Article.

