# Supplementary material for: Epifluorescence-based three-dimensional traction force microscopy
Source: Sci Rep. 2020 Oct 6;10:16599. doi: 10.1038/s41598-020-72931-6 (PMC7538907; doi:10.1038/s41598-020-72931-6)
Supplement: Supplementary file 1 — Supplementary Information. [file 41598_2020_72931_MOESM1_ESM.pdf]

# Supplemental Information for: Epifluorescence-based three-dimensional traction force microscopy

**Lauren Hazlett[1,5,+,*], Alexander K Landauer[2,5,+,*], Mohak Patel[2], Hadley A Witt[3,4], Jin Yang[5], Jonathan S Reichner[4], and Christian Franck[5,*]**

[1]Brown University, Center for Biomedical Engineering, Providence, 02912, USA
[2]Brown University, School of Engineering, Providence, 02912, USA
[3]Brown University, Pathobiology Graduate Program, Providence, 02912, USA
[4]Rhode Island Hospital, Department of Surgery, Providence, 02912, USA
[5]University of Wisconsin-Madison, Mechanical Engineering, Madison, 53706, USA
[*]cfranck@wisc.edu, lauren_hazlett@alumni.brown.edu, alexander_landauer@brown.edu
[+]these authors contributed equally to this work

## ABSTRACT

We introduce a novel method to compute three-dimensional (3D) displacements and both in-plane and out-of-plane tractions on nominally planar transparent materials using standard epifluorescence microscopy. Despite the importance of out-of-plane components to fully understand cell behavior, epifluorescence images are generally not used for 3D traction force microscopy (TFM) experiments due to limitations in spatial resolution and measuring out-of-plane motion. To extend an epifluorescence-based technique to 3D, we employ a topology-based single particle tracking algorithm to reconstruct high spatial-frequency 3D motion fields from densely seeded single-particle layer images. Using an open-source finite element (FE) based solver, we then compute the 3D full-field stress and strain and surface traction fields. We demonstrate this technique by measuring tractions generated by both single human neutrophils and multicellular monolayers of Madin-Darby canine kidney cells, highlighting its acuity in reconstructing both individual and collective cellular tractions. In summary, this represents a new, easily accessible method for calculating fully three-dimensional displacement and 3D surface tractions at high spatial frequency from epifluorescence images. We released and support the complete technique as a free and open-source code package.

## Typical processing parameters

Typical parameters and the values they have taken in our example cases (viz. rigid body displacement, synthetic validation, neutrophil and Madin-Darby canine kidney (MDCK) experiments) for deconvolution, bead localization, topology-based particle tracking (TPT), regularization and planarization, meshing and timesteps, and visualization are given in Table 1. Many of these parameter values are constant across these cases and will rarely need to be adjusted in practice (PSF size, outlier threshold), whereas the most commonly tuned of parameters are selected via iterative user prompts and informational displays or intermediately save data (threshold, minimum and maximum size, number of standard deviations, smoothness in ($x$,$y$,$z$), mesh refinement location). Others are cell-type, imaging or computational hardware related or limited (objective magnification, bead size, image sizes, mesh sizes, mesh refinement) and are often fixed in our but may need to be changed under some circumstances. A smaller selection of parameters occasionally require fine-tuning at user discretion to obtain optimal results (for example, KNN and matching parameters for TPT, element type, and quadrature degree for FEA) these algorithm set-up choices are given in Table 2 and are generally not recommended to be adjusted. An outline of the algorithm steps and recommendations for use are given in the following section, which is intended to clarify the parameter selection process.

## Procedure for computing tractions from epifluorescence images

Note that more detailed information is available in the documentation for the code at http://github.com/francklab or by contacting the authors.

### Installing necessary software packages

This package is implemented in Matlab (https://www.mathworks.com/products/matlab.html) and FEniCS (https://fenicsproject.org). Development is under Matlab 2019a on Windows 10 and requires the Statistics and Image Processing toolboxes. We have used Docker Desktop 2.2.0.5 with a custom containerized version of FEniCS[1,2] (PETSC verison 3.11.1, Dolphin 2019.1.0.post0, FEniCS 2019.1.0) available from quay.io (https://quay.io/repository/alandauer/sltfm_dev) that is downloaded automatically when first running the package via Windows PowerShell. It is also possible to use

Docker Toolbox (for older Windows and macOS versions), although this may require additional setup. Matlab and toolboxes are installed with no additional options and may be run directly. After installing Docker, the available memory should be increased in the settings pane to at or above 16GB and the storage drive with data and code should be set to shared. With these installed and configured, all data-files set up and organized (see "Data Organization" below) the Matlab run-script is run first. Once complete, use Windows PowerShell to execute the .ps1 runscript which automatically sets up, runs, and saves output data for the finite element solution. The post-processing Matlab script is then used to compute and visualize results, such as displacement

**Table 1.** Process parameters used for the complete workflow with rigid displacement and synthetic validation, neutrophil, and MDCK cell datasets.

| *Parameter* | *Rigid displacement validation* | *Synthetic validation* | *Neutrophil cell experiment* | *MDCK cell experiment* |
|---|---|---|---|---|
| **Image set up parameters** | | | | |
| Magnification | 40X (0.6 NA) | 40X (0.6 NA) | 40X (0.6 NA), 1.5X optical zoom | 40X (0.6 NA) |
| Full image size (vx) | 2560×2156×101 | 1025×1025×42 | 2560×2156×101 | 2560×2156×41 |
| Cropped image size (vx) | 1024×1024×101 | 1024×1024×42 | 1024×1024×44 | 1024×1024×41 |
| $\mu$m-per-pixel conversion ($x$,$y$) | 0.16 $\frac{\mu m}{px}$ | 0.16 $\frac{\mu m}{px}$ | 0.11 $\frac{\mu m}{px}$ | 0.16 $\frac{\mu m}{px}$ |
| z-step size ($\mu$m) | 1.0 | 0.5 | 0.3 | 1.0 |
| Bead size ($\mu$m) | 0.5 | 0.5 | 0.5 | 0.5 |
| PSF cube half size (vx) | 10 | 10 | 10 | 10 |
| **Localization parameters** | | | | |
| Threshold | 0.125 | 0.125 | 0.125 | 0.125 |
| Minimum size (vx) | 2 | 2 | 2 | 2 |
| Maximum size (vx) | 200 | 200 | 200 | 200 |
| **Tracking parameters** | | | | |
| Matching threshold | 2 | 2 | 2 | 2 |
| Outlier threshold | 5 | 7 | 7 | 7 |
| **Planarization parameters** | | | | |
| Number of standard deviations | 1.2 | 1 | 0.8 | 1.8 |
| regularization smoothness x-y | 0.00001 | 0.00001 | 0.00001 | 0.00001 |
| regularization smoothness z | 0.00001 | 0.00001 | 0.00001 | 0.00001 |
| **Finite element parameters** | | | | |
| Elastic modulus (Pa) | 1500 | 1500 | 1500 | 8300 |
| Poisson's ratio | 0.45 | 0.45 | 0.45 | 0.45 |
| Gel thickness ($\mu$m) | 70 | 70 | 70 | 70 |
| Number of steps | 10 | 10 | 10 | 10 |
| Mesh size x-y | 48 | 38 | 48 | 48 |
| Mesh size z | 18 | 18 | 18 | 18 |
| Mesh refinement factor x-y | 0.15 | 0.9 | 0.5 | 0.15 |
| Mesh refinement factor z | 0.6 | 0.6 | 0.6 | 0.6 |
| Quadrature degree | 7 | 7 | 7 | 7 |

and tractions fields.

Additional software that we have used to organize code, prepare data, visualize outputs, and generate figures includes GitHub Desktop (https://desktop.github.com) and Atom (https://atom.io), ImageJ[3] (https://imagej.net/Fiji), Paraview (https://www.paraview.org), and Illustrator (https://www.adobe.com/products/illustrator.html) or Inkscape (https://inkscape.org).

### Acquiring and saving microscopy images

Prior to imaging, bead size and density need to be selected by the user. The size and relative density of the fluorescent particles should be chosen based on the type of cell being studied, and hence the desired spatial resolution of the displacement

**Table 2.** Algorithm set-up parameters used in various steps of the workflow. These are generally not recommend to be changed, but are possible to adjust if needed.

| *Parameter* | *Default setting* |
|---|---|
| **Image set up parameters** | |
| Prefilter | Off |
| Deconvolution | Lucy-Richardson; 10 iterations |
| **Localization parameters** | |
| For loop steps | 1 |
| **Tracking parameters** | |
| KNN Feature similarity | 10 |
| KNN Feature descriptor | 16 |
| Number of spheres | 2 |
| Drift removal | ON (Note: OFF for rigid displacement experiment) |
| **Planarization parameters** | |
| Regularizer interpolant | Linear |
| Regularizer solver | Matlab "\" |
| Regularizer mesh size (px) | [1,1,1] |
| **Finite element parameters** | |
| Roll-off filter width (px) | (displacement region size)/10 |
| Roll-off filter strength (px) | (displacement region size)/50 |
| Vector function space type (displacement) | 1st order Lagrange |
| Quadrature degree | 7 |
| FFC options | optimize: True; eliminate zeros: True; precompute basis constant: True; precompute ip constant: True |
| Boundary condition interpolant | Clough-Tocher on a 5th order function space |
| Solver type | Nonlinear variational solver |
| Newton solver type | linear solver: bicgstab; preconditioner: petsc_amg; maximum iterations: 500; relaxation parameter: 1.0 |
| Newton solver tolerances | Absolute: 1E-05; Relative: 1E-05; |
| Tensor function space type (stress) | 1st order Lagrange |
| Stress projection | Solver: conjugate gradient; preconditioner: petsc_amg |
| **Traction computation parameters** | |
| Interpolation method | Natural neighbor |
| NaN removal | Laplacian interpolant |

and traction output (*viz.* smaller cells require smaller, higher density beads to resolve smaller area and amplitude substrate displacements compared to larger cells). For these experiments, 0.5 $\mu$m beads provided high enough resolution and density to reconstruct displacements from both a 10 $\mu$m neutrophil and a 100 $\mu$m cluster of MDCK cells. We recommend titrating out the bead density experimentally by diluting well-vortexed bead solution in water to find a density that does not lead to clumping, streaking, or other irregularities in bead distribution, but rather creates a uniform surface layer of beads. More information about creating polyacrylamide gels and creating a single layer of microbeads at the gel surface can be found in Pelham and Wang *et al.* 1997[4] and Knoll *et al.* 2014[5].

Table 3 contains a description of the experimental bead seeding and polyacrylamide gel surface preparation parameters used in our cellular experiment cases. This includes a measurement of the distance between the basal cell layer and the central bead plane, which gives users an estimate of how far below the cells the fiducial markers typically should reside. In addition, the table presents our computed bead density and numbers of tracked beads, which can often vary significantly across a single gel or gels prepared using the described methodology. Bead density in the single layer can easily by adjusted by altering the dilution factor of the bead solution. Measured experimental conditions, including the single layer bead spread in $z$, and bead layer roughness (see Toyjanova *et al*. 2015[6]) are also tabulated as guidelines for workable surface and bead layer characteristics.

The other main factor that influences the selection of bead size is the imaging system. Ideally, the combination of the imaging objective and camera used would, at a minimum, provide images with 3-9 pixels per bead diameter to allow TPT to fully localize the bead centers. This roughly translates to a $\mu$m-per-pixel ratio of 1/3 to 1/9 the particle diameter, or less than or equal to a 0.17 $\mu$m-per-pixel ratio for a 0.5 $\mu$m bead. Another factor to consider when selecting an objective is the presence of a correction collar: this feature allows the user to correct images for sample thickness and thus is highly recommended for TFM imaging on inverted microscopes to limit the amount of $z$-spread from fluorescent beads and improve the bead point spread function for later deconvolution.

When acquiring images, the user also needs to define a $z$-step size for capturing volumetric image sequences. We advise using the recommended setting for the microscope and objective used, if such a setting exists, or else sample approximately once per bead diameter. Image frequency can be selected by the user based upon the experimental purpose: i.e., examining changing traction patterns of fast-moving cells at short time intervals vs. probing slow-acting physiological changes at longer intervals. The final image acquired must always be a cell-free reference image of the beads alone after removing the cells from the gel surface. This needs to be done without disrupting the gel surface itself, and is commonly accomplished using a detergent, for example sodium dodecyl sulfate (SDS), to kill the cells or an enzyme such as trypsin to disrupt the cell adhesion to the surface proteins. An example of the required image quality and expected visible bead displacements from our MDCK example case can be seen in Fig. 1b.

To interface with our code, all final bead images need to be converted to .tif files, with each fluorescence channel, XY location (i.e., multipoint), and time interval (i.e., timepoint) of data saved as its own .tif stack. Most microscope acquisition software will allow users to convert to .tif files, and ImageJ can also be used to convert microscopy images to .tifs. Users may want to crop .tif files prior to being input into the code, which is possible to do using either the microscope software or ImageJ.

**Table 3.** Experimental bead seeding and single layer surface parameters for neutrophils and MDCK cells.

| *Parameter* | *Neutrophil* | *MDCK* |
|---|---|---|
| **Surface fabrication** | | |
| Bead solution dilution ratio | 1:100 | 1:100 |
| Surface functionalization | Sulfo-SANPAH | Sulfo-SANPAH |
| Surface protein coating | Human plasma fibronectin | Rat-tail collagen type-I |
| Cell basal surface to bead center plane (est., $\mu$m) | 4.1 ± 0.91 | 6.3 ± 0.85 |
| **Bead layer parameters** | | |
| Tracked beads in ROI (timepoint 1 / timepoint 2) | 3734 / 3846 | 2511 / 2488 |
| Seeding density (beads/$\mu$m$^2$) (timepoint 1 / timepoint 2) | 0.29 / 0.30 | 0.094 / 0.093 |
| Bead z-spread ($\mu$m) | -0.08 ± 0.47 | -0.02 ± 0.36 |
| Bead layer average roughness $R_a$ ($\mu$m) | 0.52 | 0.21 |

### Processing options for cell outlines

Our code package does not provide a cell segmentation tool, both because the cell outlines are used for primarily visualization purposes and because there exist many such tools that perform admirably for epifluorescence images and are straightforward to use. Three-dimensional image stacks of fluorescently labeled cells can be captured using a fluorescence channel if it is possible to label the cells and capture these images. If phototoxicity is a concern or if the cells can not be labeled, a single brightfield, phase contrast, or epifluorescence image can be acquired at the plane of maximum cell spread. For three-dimensional fluorescence images, we recommend converting the image files to .mat files and performing image segmentation in Matlab using built-in thresholding and segmentation tools. These same Matlab built-in tools can be used for segmenting phase contrast or brightfield images, but these images tend to be more difficult to segment due to background noise from the beads directly below the cell, and thus some users might find it easier to segment cells using ImageJ, which has several available plug-ins for image segmentation, including options to segment images by hand. For reference on how to segment images in Matlab, see: `https://www.mathworks.com/help/images/detecting-a-cell-using-image-segmentation.html`. For reference on how to segment images using ImageJ, see: `https://imagej.net/Segmentation`. An example of the expected image quality for acquired fluorescence images of cells is shown in Fig. 1a.

The cell images must be cropped to the exact same specifications (i.e., size and location) as the final bead image files. The final format for cell images required for our visualization codes is a .mat file containing a separate 3D matrix of binarized (black and white) cell image for each multipoint and timepoint of data. In the event that only a single cell image was captured, rather than an image stack, a .mat file containing the single binarized image for each multipoint and timepoint is also acceptable.

### Data organization

To set up the data files for processing through our code package, all files must be set up in the expected file structure within Matlab's file path. We recommend storing the entire code package in its original folder and adding a subfolder to contain the data for ease of access and pathing. The "`data`" folder should contain the segmented cell .mat file and a separate "`tifs`" subfolder, which should in turn contain a separate subfolder for each multipoint of data. Each multipoint subfolder should contain the .tif stack for each timepoint of data, preferably with a naming format that will ensure Matlab reads the files in the proper order. We suggest creating file names ending in numbers, (i.e., '000', '001', etc. or 't01', 't02'), where the first image should be the reference (undeformed, cell-free) image. The code will automatically generate additional subfolders within the "`data`" folder to contain the converted .mat files, the user-selected PSFs, deconvolved .mat files, and figures produced; each of which will contain a separate subfolder for each multipoint of data.

Multipoint processing capability was created with the intention of processing data for direct comparison at the same time, using the same processing parameters. For example, multiple cells from a single gel can be imaged simultaneously and then processed through Matlab together, or data from multiple gels with different experimental conditions can be compared statistically when processed using the same parameters.

### Computing and regularizing displacements

Within the Matlab runfile, bead image .tif files are automatically converted to .mat files, cropped to user specifications if desired, and then the code prompts users to select the center of a single bead to be used as the point spread function for deconvolution. The deconvolved bead .mat files are then run through the topology-based particle tracking (TPT) code[7]. The code prompts users to confirm or change the pre-set localization parameters for TPT, including bead size and thresholding. Users can also alter the pre-set tracking parameters for TPT, including the feature descriptor, matching threshold, and outlier threshold. We advise users keep the same tracking parameters for all data sets intended for statistical comparison, and advise that users begin with the pre-set TPT parameters, as changes to these parameters can significantly change the tracking results. The code will display the final tracking and displacement results to the user for approval. Following particle tracking, the 3D displacement of each individual particle is computed, a step which can be done incrementally by comparing each timepoint to the previous one, or cumulatively by comparing each timepoint to the reference image. For more details, see the user guide on GitHub or contact the authors.

The initial displacement computation is performed on scattered data and is planarized to fit the finite element boundary condition. The first step in this process is user-defined bead outlier removal: the code displays the spread of localized beads from the experimental images and the user inputs the desired removal parameter, which is a number of standard deviations from the mean bead $z$-location. The final bead locations are used to fit the top surface plane, onto which the displacements are then regularized using Jason Nicholson's regularizeNd (see `https://github.com/jasonnicholson/regularizeNd`) package. This regularization scheme allows the user to tune the displacement smoothing parameter. The code outputs a plot of displacement magnitudes smoothed with the default smoothing parameter along with the initial scattered displacement data and prompts the user to alter the smoothing parameter if desired. This visualization method is meant to prevent over- or under-smoothing of the data. We recommend users use the same smoothing parameters for comparable data sets, unless

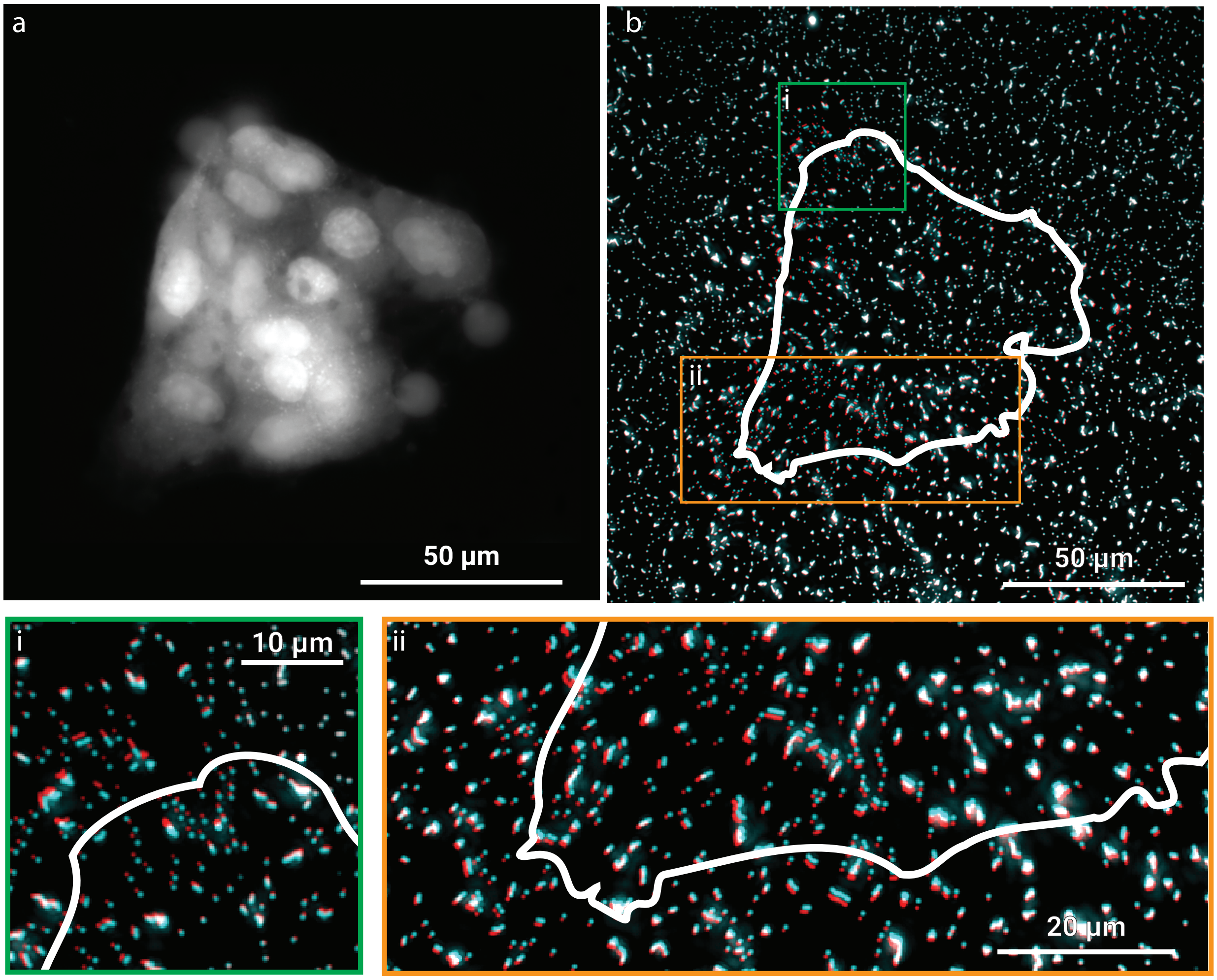


**Figure 1.** Epifluorescence images of MDCK cells and underlying beads to show expected image quality. (a) Epifluorescence image acquired of Calcein-AM labeled MDCK cell cluster on PA at a single plane at maximum cell spreading. (b) Maximum intensity projections of the beads in the PA gel underneath the MDCK cell cluster in the reference or cell-free (shown in magenta) and deformed (shown in cyan) images. Where there is no difference between the bead locations in the reference and deformed image, the beads show up as white. An outline of the MDCK cell cluster, generated using a Matlab-based image thresholding method, is shown at its respective location on the beads for reference. (i) and (ii) show the areas within the green and orange boxes enlarged to show detail.

experimental conditions produce a significant change in bead scatter or displacement magnitude and thus requires different smoothing considerations (*e.g.* a cell treatment that affects the actin/myosin structure).

### Reconstructing traction fields using finite element analysis

Once the Matlab runfile has completed, a sl_tfm_call_*.py script file for each multipoint, based on the template sl_tfm_call.py file, are written to the main code directory. In addition, an input .mat datafile for each multipoint and timepoint is saved to the main code directory. With these generated, Windows PowerShell is used to run the FEA: "cd" to the main code directory, run "`Set-ExecutionPolicy -Scope CurrentUser RemoteSigned`" and confirm, then run the FEA package script via "`.\run_sl_tfm_ps.ps1`". A series of output prompts culminating in "`All done!`" are printed. Intermediate files containing the generated mesh are storied in the .pvd+.vtu format. Results files in both .mat and .pvd+.vtu are saved out to the main code directory. Once FEA completes in PowerShell, return to Matlab and open the post-FEniCS post-processing script. Update input configuration information at the top of the file (i.e., multipoint names, etc) and run to extract tractions from the deformed surface and stresses from FEniCS (output will be read automatically).

Within this process there are several parameters that may (optionally) be adjusted. First, the number of load steps used to

compute the solution is entered in the Matlab runscript – fewer load steps reduce the computational cost, but may lead to poor convergence and values between 3 and 15 are typical. In the Python code used to set up and solve the FEniCS several meshing parameter can be adjusted: number of elements in ($x$,$y$,$z$) where fewer elements will give poorer results but reduce computational costs (RAM and time) – our test cases reached reasonable convergence with approximately (38,38,18). In addition, the mesh is refined by a power-law mapping in the $z$-direction (typically 0.6) and exponentially (Gaussian-like) mapping independently in $x$- and $y$-directions (typically $\sigma\_ = 0.1$ to $\sigma\_ = 0.5$) about the centroid of the cell, which is computed directly from the cell image file. Quadrature degree for the hexahedron elements may also be adjusted if needed. Intermediately saved mesh files and output stresses or displacements may be directly visualized in Paraview (version 5.6.x, newer versions have introduced bugs when reading Dolphin-written .pvd+.vtu files). Note that .pvd+.vtu are written and read with directly sampled nodal values, rather than projecting to quadrature points, and thus only approximate the results and tend to exaggerate mesh artifacts.

### Plotting displacement and traction fields

Displacement and traction magnitude plots akin to those shown in Fig. 4(a-d)(i) in the main text are automatically produced and saved by the code. The figures contain a contour plot of a maximum intensity projection of the cell image file, along with a coneplot of the 3D displacement or traction magnitude. These figures are automatically cropped to a user-defined area and size, and the cone size and relative density are also tunable by the user for optimal visualization. The parameters used to crop and plot the displacement and traction magnitude of the neutrophil and MDCK cell shown in Fig. 4(a-d)(i) of the main text are found in Table 4.

The choice for the type of plots to use for traction data visualization is ultimately dictated by personal preference of the experimenter and the type of scientific questions to be answered. While we provide the code required to create the coneplots shown in Fig. 4 of the main text, contour plots are also often chosen to display cell displacement and tractions. It is relatively straightforward for users to plot contour maps from the displacement and traction data output from our code. An example of traction contour maps from our example neutrophil and MDCK cell cases, including individual plots for $x$, $y$, $z$, and $xyz$-magnitudes can be seen in Fig. 2.

**Table 4.** Parameters used for plotting displacement and traction magnitude of neutrophils and MDCK cells.

| *Parameter* | *Neutrophil displacement plot* | *Neutrophil traction plot* | *MDCK displacement plot* | *MDCK traction plot* |
|---|---|---|---|---|
| **Image cropping** | | | | |
| Cropped image size (vx) | 1024×1024×44 | 1024×1024×44 | 1024×1024×41 | 1024×1024×41 |
| Figure size (px) | 270×280 | 270×280 | 920×915 | 920×915 |
| Figure crop $x$-min and max (px) | 380, 650 | 380, 650 | 50, 970 | 50, 970 |
| Figure crop $y$-min and max (px) | 350, 630 | 350, 630 | 65, 980 | 65, 980 |
| **Visualization Parameters** | | | | |
| Cone size | 0.008 | 0.008 | 0.012 | 0.025 |
| Seeding density | 2.5 | 2.5 | 6 | 6 |

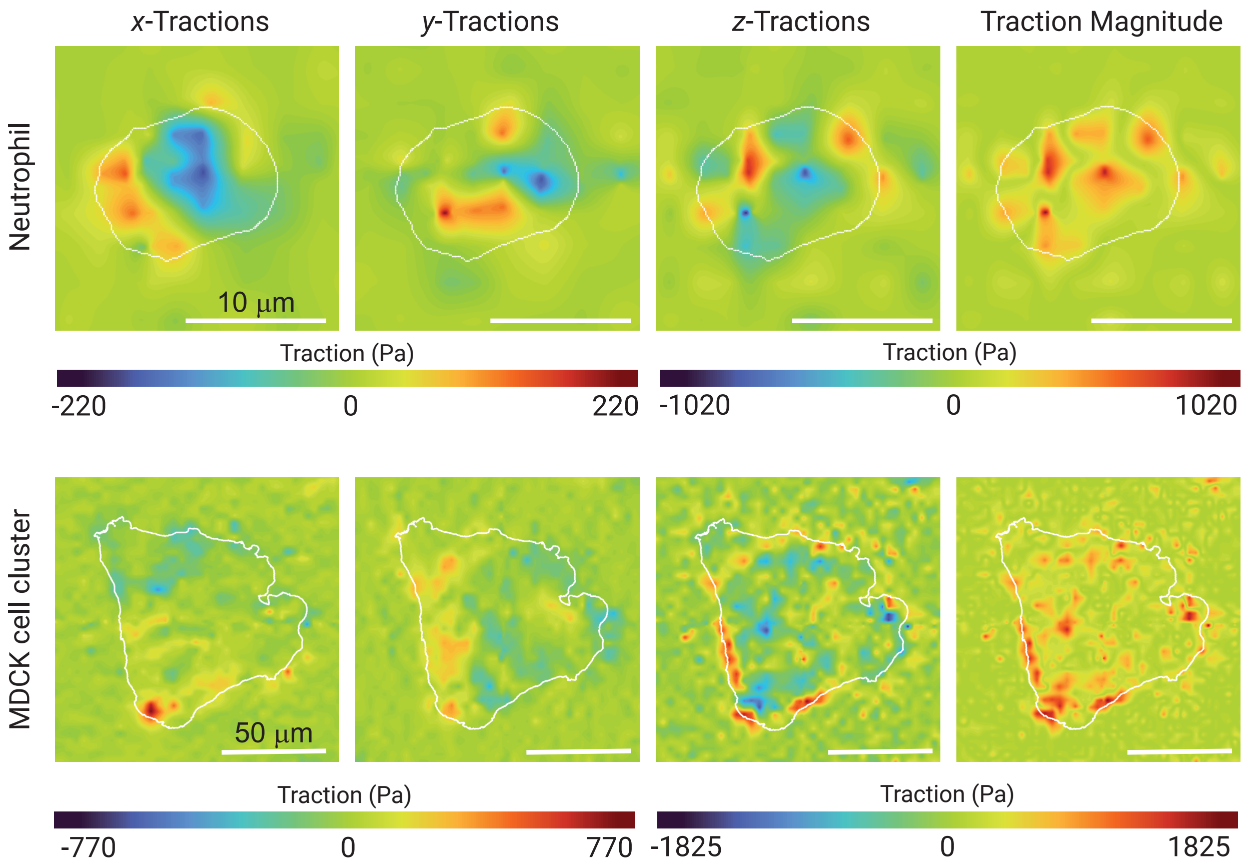


**Figure 2.** Contour plots of neutrophil (top row) and MDCK cell cluster (bottom row) tractions, showing the $x$, $y$, $z$, and $xyz$-magnitudes. Cell or cell cluster outlines are shown in white. Neutrophil scale bar across all images: 10 μm. MDCK cell scale bar across all images: 50 μm.

## References


**1.** Alnæs, M. *et al.* The FEniCS project version 1.5. *Arch. Numer. Softw.* **3** (2015).

**2.** Logg, A., Mardal, K.-A. & Wells, G. *Automated solution of differential equations by the finite element method: The FEniCS book*, vol. 84 (Springer Science & Business Media, 2012).

**3.** Schindelin, J., Rueden, C. T., Hiner, M. C. & Eliceiri, K. W. The ImageJ ecosystem: An open platform for biomedical image analysis. *Mol. Reproduction Dev.* **82**, 518–529, DOI: 10.1002/mrd.22489 (2015).

**4.** Pelham, R. J. & Wang, Y.-l. Cell locomotion and focal adhesions are regulated by substrate flexibility. *Proc. Natl. Acad. Sci.* **94**, 13661–13665, DOI: 10.1073/pnas.94.25.13661 (1997).

**5.** Knoll, S. G., Ali, M. Y. & Saif, M. T. A. A novel method for localizing reporter fluorescent beads near the cell culture surface for traction force microscopy. *J. Vis. Exp. : JoVE* 51873, DOI: 10.3791/51873 (2014).

**6.** Toyjanova, J., Flores-Cortez, E., Reichner, J. S. & Franck, C. Matrix confinement plays a pivotal role in regulating neutrophil-generated tractions, speed, and integrin utilization. *J. Biol. Chem.* **290**, 3752–3763 (2015).

**7.** Patel, M., Leggett, S., Landauer, A., Wong, I. & Franck, C. Rapid, topology-based particle tracking for high-resolution measurements of large complex 3d motion fields. *Sci. Reports* **8**, 5581, DOI: 10.1038/s41598-018-23488-y (2018).
